# Depression, anxiety and stress among Swedish university students before and during six months of the COVID-19 pandemic: A cohort study

**DOI:** 10.1177/14034948211015814

**Published:** 2021-05-26

**Authors:** Fred Johansson, Pierre Côté, Sheilah Hogg-Johnson, Ann Rudman, Lena w. Holm, Margreth Grotle, Irene Jensen, Tobias Sundberg, klara Edlund, Eva Skillgate

**Affiliations:** 1 Musculoskeletal & Sports Injury Epidemiology Center, Department of Health Promotion Science, Sophiahemmet University, Sweden; 2 Unit of Intervention and Implementation Research for Worker Health, Karolinska Institutet, Sweden; 3 Faculty of Health Sciences and Centre for Disability Prevention and Rehabilitation, Ontario Tech University, Canada; 4 Canadian Memorial Chiropractic College, Dalla Lana School of Public Health, University of Toronto, Canada; 5 Department of Clinical Neuroscience, Karolinska Institutet, Sweden; 6 School of Education, Health and Social Studies, Dalarna University, Sweden; 7 Department of Physiotherapy, Faculty of Health Sciences, Oslo Metropolitan University, Department of Physiotherapy, Norway; 8 Research and Communication Unit for Musculoskeletal Disorders (FORMI), Oslo University Hospital, Norway

**Keywords:** Depression, anxiety, stress, mental health, COVID-19, coronavirus, students, Sweden

## Abstract

**Aims:**

The COVID-19 pandemic has had a profound effect on societies and citizens worldwide, raising concerns about potential mental health impacts. We aimed to describe trajectories of depression, anxiety and stress symptoms during the COVID-19 outbreak compared to before the outbreak, and to determine if trajectories were modified by pre-pandemic loneliness, poor sleep quality and mental health problems.

**Methods:**

We conducted a cohort study with 1836 Swedish university students entering the study before 13 March 2020, the onset of the pandemic, with follow-ups within three (FU1) and six months (FU2) of the outbreak. Generalized Estimating Equations were used to estimate mean differences in symptom levels over time-periods, and to estimate potential effect modifications.

**Results:**

We found small differences in mean levels of the depression, anxiety and stress scale (DASS-21) over time. Compared to before the pandemic, depression increased by 0.25 points of 21 (95% CI: 0.04 to 0.45) at FU1 and decreased by 0.75/21 (95% CI:−0.97 to −0.53) at FU2. Anxiety decreased from baseline to FU1 by 0.09/21 (95% CI: −0.24 to 0.07) and by 0.77/21 (95% CI: −0.93 to −0.61) to FU2. Stress decreased from baseline to FU1 by 0.30/21 (95% CI: −0.52 to −0.09) and by 1.32/21 (95% CI: −1.55 to −1.09) to FU2. Students with pre-pandemic loneliness, poor sleep quality or pre-pandemic mental health problems did not have worse trajectories of mean mental health symptoms.

**Conclusions:**

Symptom levels were relatively stable during the first three months of the pandemic, while there was a slight decrease during the summer months, probably due to seasonality effects.

## Background

The COVID-19 pandemic has had a profound impact globally on societies and the daily lives of citizens. In a call for action, Holmes et al. [1] raised concern about the detrimental effects the pandemic might have on mental health and urgently called for research to evaluate its impact on mental health. Elevated mental health problems among university students have been well documented prior to the COVID-19 pandemic [2, 3]; therefore, it is of utmost importance to investigate the impact of the pandemic on mental health among this already vulnerable population.

A ‘living’ systematic review of the literature found that most studies reported small to negligible worsening of mental health symptoms during the pandemic [4]. However, data from a large UK cohort study suggest that levels of depression and anxiety decreased in the first few months of the lock-down [5]. For university students, some studies report increasing depression and anxiety during COVID-19 [6, 7], while some reported slight decreases [8].

The impact of physical distancing and loneliness on mental health during the COVID-19 pandemic has been expressed as a key concern [9]. Loneliness is a predictor for the development of depression [10] and is associated with worse prognosis for depressed individuals [11]. Living alone has been reported as a risk factor for depression and anxiety during the COVID-19 pandemic [12]. Similarly, poor sleep quality is a prevalent and increasing problem among university students [13], which has also been associated with depression, anxiety and stress during the COVID-19 pandemic in the general population [14]. Physiologically, poor sleep quality impairs emotional regulation and increases affective reactivity [15], and further is associated with increased negative emotions following disruptive events [16]. As highlighted by Yao et al. [17], individuals with pre-existing mental health problems may predispose individuals to experience worse mental health outcomes during the pandemic. Two cross-sectional studies suggest increased symptoms of anxiety and eating disorders as well as other psychiatric symptoms among psychiatric patients during the COVID-19 pandemic [12]. This may be significant also for individuals with minor mental health problems because they are at risk for developing more severe problems [18, 19].

In Sweden, the spread of COVID-19 accelerated rapidly beginning in early March 2020. On 13 March 2020, the Public Health Agency of Sweden declared that the risk of community-acquired COVID-19 had reached a very high level and issued recommendations to stay at home when experiencing symptoms [20]. Four days later, on 17 March, universities cancelled all campus-based education and switched to online education [21]. Like in many other countries, Sweden’s public health strategy to control the spread of the virus focused on promoting physical distancing, strongly encouraging reductions in social contacts, mobility and travelling. Population-based surveys indicate that most of the Swedish population adhered to these recommendations during the spring of 2020 [22]. However, unlike in many other countries, no enforced general lockdowns have yet been issued. Compared to the other Scandinavian countries, Sweden has had a high COVID-19 mortality, and the government’s strategy to contain the spread of the virus has received international attention.

Given Sweden’s unique strategy, and the initiation of the Sustainable University Life (SUN-study) in August 2019, there is a unique opportunity to conduct a longitudinal evaluation of the mental health impact of the COVID-19 pandemic among Swedish university students. University students, with generally higher levels of mental health problems than the general population [3], are a potential at-risk group during the COVID-19 pandemic. Additionally, students with pre-pandemic loneliness, poor sleep and poor mental health may be at even higher risk.

We aimed to describe the trajectories of depression, anxiety and stress symptoms in Stockholm university students before and during the first six months of the COVID-19 pandemic. Our secondary aim was to determine whether trajectories of depression, anxiety and stress symptoms were modified by pre-pandemic loneliness, poor sleep quality or mental health problems. We hypothesized that symptoms of depression, anxiety and stress would worsen during the COVID-19 pandemic, and that the worsening would be more severe for students with loneliness, poor sleep quality and pre-pandemic mental health problems.

## Methods

### Design and study population

We conducted a cohort study of university students in Stockholm, Sweden with recruitment beginning before the outbreak of COVID-19 in Sweden and follow-up surveys at three-month intervals continuing after the outbreak. This cohort study is nested within a larger dynamic cohort study of university students: the Sustainable University Life (SUN-study) (http://clinicaltrials.gov/ ID: NCT04465435), in which data collection began in August 2019 and will be ongoing until November 2021.

Participants were recruited from six universities in Stockholm, covering medical, economic, technical and sport science education programs. The targeted universities were selected due to practical and financial limitations. Full-time undergraduate students with at least one year left to complete their degree were eligible for inclusion. Students who completed the baseline questionnaire before 13 March 2020 were included in the study reported here.

### Data collection

Students received information about the study through in-class presentations by study staff. Access links to the study questionnaire were distributed via email. Information about the study was also provided on relevant social media channels (e.g. student union social media channels) and at on-campus information sites. Participants were followed via web surveys every three months for one year and chose to fill out the survey either in Swedish or English. Participants not responding to follow-up surveys received reminders by email, text-message and one phone call. The study was approved by the Swedish Ethical Review Authority (reference number: 2019-03276, 2020-01449). Informed consent was provided by all participants electronically prior to completing the baseline survey.

### Data organization

**Figure 1.**
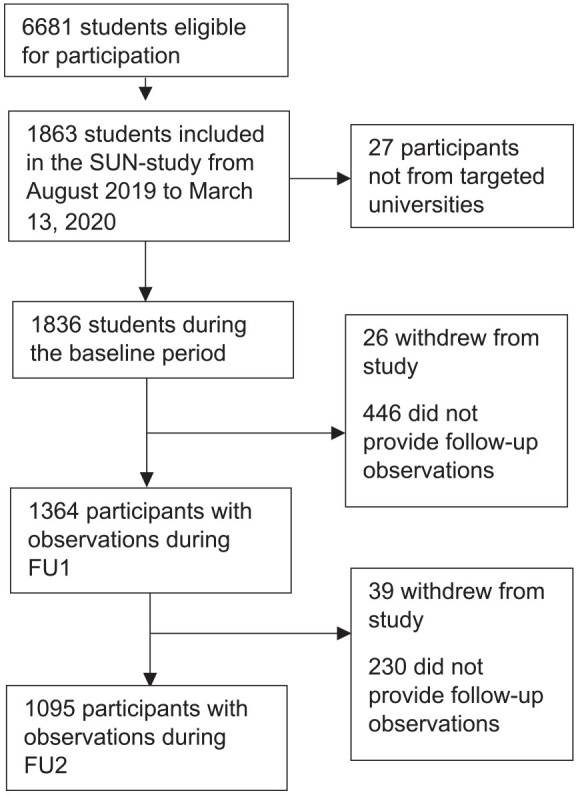
Flow chart of inclusion of participants.

The collected data was divided into three time-periods: before the pandemic (19 August 2019 to 13 March 2020), follow-up period 1 (FU1; 14 March to 15 June 2020) and follow-up period 2 (FU2; 16 June to 10 September 2020) (Figure 1, Supplemental eFigure 1). The date marking the start of the pandemic, 13 March, is the date that the Public Health Agency of Sweden declared that Sweden had entered a new phase in the pandemic with high risk of community acquired COVID-19 [20]. Four days later, 17 March, Sweden closed all on-campus educational activities at universities, transitioning to on-line education. The cut-off point for FU2, 15 June, was selected because most university semesters had ended by then (eFigure 1).

### Outcomes

Symptoms of depression, anxiety and stress were measured with the short-form depression, anxiety and stress scale (DASS-21) [23] at each time-period. DASS-21 consists of 21 items rated on a 4-point scale ranging from 0 (Did not apply to me at all) to 3 (Applied to me very much, or most of the time). Scores for the depression, anxiety and stress subscales are the sums of the 7 subscale-specific items yielding scores ranging from 0 to 21 with higher scores indicating more severe symptoms. DASS-21 has good psychometric properties, with evidence of convergent validity for all subscales, and reported Cronbach’s α values of 0.77 to 0.92 for the three subscales [23, 24].

### Exposures

Pre-pandemic mental health problems were classified at baseline as scoring above the recommended DASS-21 cut-offs for moderate symptom levels on any of the three subscales (⩾ 7 on the depression subscale, ⩾ 6 on the anxiety subscale, ⩾ 10 on the stress subscale) [25]. These cut-offs are slightly higher than those found to discriminate between a normal population and an outpatient psychiatric population [26].

Pre-pandemic loneliness was measured using the UCLA three-item loneliness scale (UCLA-3) [27], with items rated from 1 (Hardly ever) to 3 (Often). Items are summed to give a score with potential range from 3 to 9. In accordance with previous research, we used a cut-off of ⩾ 6 to define loneliness [28]. The scale has acceptable internal consistency (Cronbach’s α = 0.72) and high correlation (*r* = 0.82) with the 20-item revised UCLA loneliness scale [27].

We used the Pittsburgh sleep quality index (PSQI) to measure pre-pandemic sleep quality [29]. The PSQI includes 19 items covering seven domains of sleep quality with component scores ranging from 0 to 3. The global score is a sum of the seven component scores ranging from 0 to 21(higher scores suggest poorer sleep quality). A global score of > 5/21 was used to classify poor sleep quality. This cut-off has sensitivity 89.6% and specificity 86.5% for differentiating between good and poor sleepers [30]. The PSQI has adequate internal consistency (Cronbach’s α = 0.82) and test–retest reliability (*r* = 0.82) over one month [30].

### Statistical analyses

We used generalized estimating equations (GEE) to model mental health symptoms over three time periods. GEE models treat correlation between observations from the same individual as nuisance parameters and provide estimates of marginal population means of the outcome. Our data was not normally distributed, which was one reason for choosing GEE since these models do not rely on the assumption of normally distributed outcome measures or the normality of residuals. We built three separate models, one each for symptoms of depression, anxiety and stress, to assess overall mean differences in symptoms over the three time periods. These models included only time-period as the predictor in order to estimate mean differences in symptom levels from baseline across follow-up periods for the full group. All GEE models, including the models described below, were specified with exchangeable working correlation structures, robust sandwich variance estimators and Gaussian link functions.

Subsequently nine separate models were fitted to assess differences in the trajectories over time by exposure levels. We dichotomized our exposures (loneliness, poor sleep quality and pre-pandemic mental health problems), into exposed and unexposed groups as described above. An interaction term between exposure and time-period was included, letting the mean symptom levels over time to vary between exposed and unexposed. This allows estimation of difference-in-differences between mean symptom levels among exposed/unexposed over time. These models were adjusted for age and gender, which were selected a priori based on previous literature.

We conducted sensitivity analyses to examine potential attrition bias by performing complete case-analyses (using data from participants who provided complete follow-up). Furthermore, we conducted a sensitivity analysis in a sample of 496 participants with baseline assessment in August to September 2019 and first follow-up prior to the pandemic in November 2019 to January 2020 to compare the trajectories of exposed and unexposed groups during the pandemic to those of an earlier time-period. Graphs from these sensitivity analyses were compared to those from the main analysis by visual inspection.

Cronbach’s α values for the scales used were constructed from the baseline data of the cohort to determine their internal consistency in our cohort.

Mean imputation by the individual scale means was used to handle missing data for the PSQI that arose due to initial technical problems with the web survey. Three items of the scale were missing (5b, 5f and 5j) for the first 512 respondents [30]. No other variables on any scales had missing values.

All analyses were performed using RStudio ver-sion 1.2.5001, the packages ‘geepack’ ‘psych’ and ‘emmeans’ were used to perform GEE analyses and to derive estimated marginal means from the models.

## Results

We invited 6681 students and 1836 (27%) agreed to participate. Of those, 74% (*n* = 1364) and 60% (*n* = 1095) provided follow-up data during the first and second follow-up periods respectively (Figure 1 and Supplemental eFigure 1). At baseline, respondents had a mean age of 26.5 years (SD 6.8), 73% were female and 80% were born in Sweden (Table I). Cronbach’s alpha values for baseline measures in our sample were 0.91, 0.79 and 0.87 for the DASS-21 subscales depression, anxiety and stress, 0.83 for the UCLA-3 and 0.75 for the PSQI.

**Table I.** Participants baseline characteristics for all and for students only participating in follow-up periods 1 (FU1) and 2 (FU2) respectively.

	All participants (*n* = 1836)	Participants followed at FU1 (*n* = 1364)	Participants followed at FU2 (*n* = 1095)
DASS-21 Depression, mean (SD)	4.6 (4.8)	4.6 (4.6)	4.3 (4.5)
DASS-21 Anxiety, mean (SD)	3.2 (3.5)	3.1 (3.5)	3.0 (3.3)
DASS-21 Stress, mean (SD)	6.5 (4.6)	6.5 (4.6)	6.3 (4.6)
Age, mean (SD)	26.5 (6.8)	26.8 (7.0)	27.0 (7.1)
Females, *n* (%)	1358 (73)	1033 (76)	837 (76)
Type of education, *n* (%)			
Medical	1596 (87)	1204 (88)	974 (89)
Economics	119 (7)	75 (6)	51 (5)
Technical	80 (4)	54 (4)	43 (4)
Sports science	41 (2)	31 (2)	27 (2)
Lonely, *n* (%)	696 (37)	506 (37)	399 (36)
Moderate mental health problems, *n* (%)	721 (39)	533 (39)	406 (37)
Poor sleep quality, *n* (%)	1024 (60)	764 (56)	586 (54)
Year of study, *n* (%)			
First	910 (50)	624 (46)	490 (45)
Second	253 (14)	204 (15)	163 (15)
Third	306 (16)	235 (17)	196 (18)
Masters	367 (20)	301 (22)	246 (23)
At least one parent with university education, *n* (%)	1300 (70)	970 (71)	778 (71)
Country of origin, *n* (%)			
Sweden	1489 (80)	1133 (83)	908 (83)
Scandinavia	91 (5)	67 (5)	58 (5)
Europe	88 (5)	45 (4)	45 (4)
Outside Europe	168 (9)	84 (8)	84 (8)

DASS-21, depression, anxiety and stress scale.

**Figure 2.**
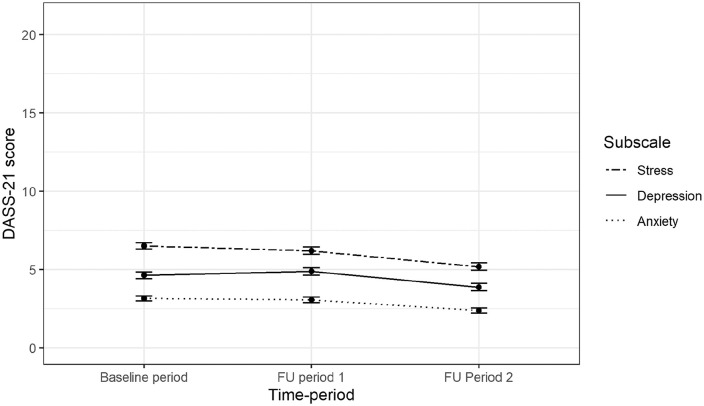
Mean scores on the depression, anxiety and stress scale (DASS-21) subscales over the three time periods.

We compared symptom levels during FU1 and FU2 to baseline levels, for the three DASS-21 subscales (Table II first row, Figure 2). Mean depressive symptom levels were 0.25/21 points higher at FU1 (mean difference 0.25 (95% CI: 0.04 to 0.45)) and 0.75/21 points lower at FU2 (mean difference −0.75, (95% CI: −0.97 to −0.53)). Mean anxiety levels were 0.09/21 points lower at FU1 (mean difference −0.09 (95% CI: −0.24 to 0.07)), and 0.77/21 points lower at FU2 (mean difference −0.77 (95% CI: −0.93 to −0.61)). Mean stress symptom levels were 0.30/21 points lower at FU1 (mean difference −0.30 (95% CI: −0.52 to −0.09)) and 1.32/21 points lower at FU2 (mean difference −1.32 (95% CI: −1.55 to −1.09)), compared to baseline levels. The estimates for differences between time-periods from the complete-case sensitivity analyses were with-in 0.13 points from the original estimates (Supplemental eTable I).

**Table II.** Generalized estimating equations’ model coefficients on mean level depression, anxiety and stress symptoms over the three time periods.

	Depression coefficients (95% CI)	Anxiety coefficients (95% CI,)	Stress coefficients (95% CI)
Models with only time periods			
Intercept	4.64 (4.42 to 4.85)	3.15 (2.99 to 3.31)	6.51(6.30 to 6.72)
FU1	0.25 (0.04 to 0.45)	−0.09 (−0.24 to 0.07)	−0.30 (−0.52 to −0.09)
FU2	−0.75 (– 0.97 to −0.53)	−0.77 (−0.93 to −0.61)	−1.32 (−1.55 to −1.09)
Models with loneliness and time period			
Intercept	3.74 (3.02 to 4.46)	2.34 (1.79 to 2.89)	4.59 (3.83 to 5.35)
Loneliness	4.22 (3.77 to 4.66)	2.34 (1.79 to 2.89)	3.21 (2.80 to 3.63)
FU1	0.55 (0.32 to 0.79)	0.16 (−0.03 to 0.33)	−0.02 (−0.28 to 0.23)
FU2	−0.29 (−0.54 to −0.04)	−0.43 (−0.61 to −0.26)	−1.14 (−1.41 to −0.87)
Loneliness * FU1	−0.79 (−1.23 to −0.34)	−0.65 (−0.98 to −0.31)	−0.76 (−1.22 to − 0.30)
Loneliness * FU2	−1.23 (−1.71 to −0.74)	−0.90 (−1.27 to −0.54)	−0.50 (−1.01 to 0.00)
Models with sleep quality and time period			
Intercept	3.31 (2.58 to 4.04)	1.94 (1.39 to 2.49)	3.85 (3.10 to 4.61)
Poor sleep quality	3.83 (3.45 to 4.21)	2.45 (2.17 to 2.74)	3.76 (3.39 to 4.61)
FU1	0.57 (0.31 to 0.83)	0.20 (−0.00 to 0.39)	0.06 (−0.25 to 0.37)
FU2	−0.22 (−0.47 to 0.03)	−0.34 (−0.53 to −0.15)	−0.80 (−1.10 to −0.50)
Poor sleep quality * FU 1	−0.58 (−0.98 to −0.18)	−0.51 (−0.81 to −0.21)	−0.68 (−1.11 to −0.26)
Poor sleep quality * FU2	−0.98 (−1.41 to −0.55)	−0.75 (−1.07 to −0.44)	−0.99 (−1.44 to −0.53)
Models with PPMHP and time period			
Intercept	2.12 (1.55 to 2.69)	1.10 (0.65 to 1.54)	2.73 (2.10 to 3.35)
PPMHP	6.90 (6.52 to 7.27)	4.58 (4.28 to 4.88)	6.59 (6.26 to 6.92)
FU1	1.15 (0.94 to 1.35)	0.51 (0.36 to 0.66)	0.58 (0.34 to 0.81)
FU2	0.32 (0.11 to 0.53)	0.04 (−0.10 to 0.18)	−0.32 (−0.55 to −0.08)
PPMHP * FU1	–2.30 (–2.74 to −1.86)	−1.54 (−1.88 to −1.20)	–2.28 (–2.73 to −1.82)
PPMHP * FU2	–2.78 (–3.28 to −2.28)	–2.12 (–2.48 to −1.75)	–2.63 (–3.14 to −2.12)

All models, except the ones with only time as predictor, were adjusted for gender (female v. male and other) and age (continuous scale).

FU1, first follow-up period; FU2, second follow-up period; PPMHP, pre-pandemic mental health problems.

**Figure 3.**
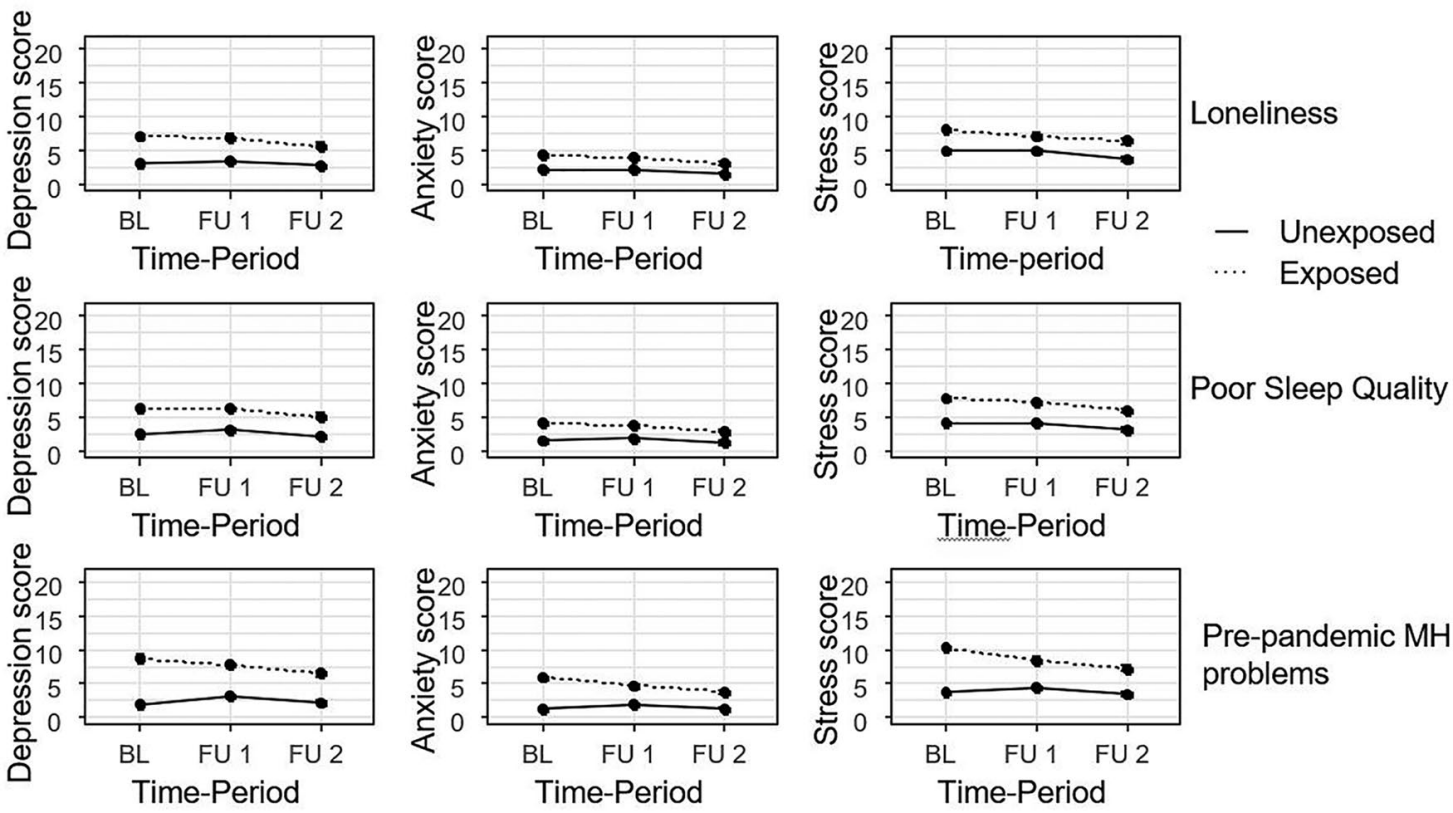
Estimated mean of depression, anxiety and stress scale (DASS-21) scores over time stratified by loneliness, sleep quality and pre-pandemic mental health (MH) problems. Adjusted for gender and age. BL, baseline.

Participants exposed to pre-pandemic loneliness, poor sleep quality or mental health problems all had higher mean levels of depression, anxiety and stress before the pandemic (Table II, Figure 3). At FU1 and FU2, mean differences between the exposed and unexposed were all smaller than before the pandemic, with none of the confidence intervals spanning zero indicating different trajectories between exposed and unexposed (Table II lower rows, Figure 3).

The difference-in-difference estimates from the complete-case sensitivity analyses comparing exposed and unexposed were all within 0.21 points of the original analyses and all in the same direction as in the main analyses (Supplemental eTable I).

Results from sensitivity analyses in the sample of 496 participants with pre-pandemic first follow-up between November 2019 and January 2020 are displayed in Supplemental eFigure 2. Visual comparison of these results with those presented in Figure 2 show similar trajectories.

## Discussion

We investigated differences in symptoms of depression, anxiety and stress in Swedish university students comparing levels before the outbreak of COVID-19, during the first months of the pandemic when the virus was spreading rapidly (14 March to 15 June 2020) and during the following three summer months (16 June to 10 September 2020) when the rate of spread of the virus had declined but by no means stopped. Our hypothesis that symptoms of would worsen during the pandemic was not supported by our data. Symptom levels were relatively stable during the first three months of the pandemic compared to pre-pandemic levels, with only a slight increase in depressive symptoms. During the following three summer months symptoms levels decreased, which we believe may be related to seasonality effects and changes in everyday life brought about by the summer vacations. However, it may also be related to the decreased spread of COVID-19 in Sweden; it is not possible to separate out these two explanations with the current data and design. Nevertheless, all these group-level changes were small in relation to what would be considered clinically relevant change on an individual level [26].

We hypothesized worse mental health trajectories for groups of students who were lonely, had poor sleep quality or pre-pandemic mental health problems. Our data did not support this hypothesis either. During the pandemic, the exposed groups showed relatively favourable trajectories of mental health symptoms compared to the unexposed groups, with larger decreases in mental health symptoms for the exposed groups as compared to the unexposed groups. These patterns are similar to those seen among students in the SUN cohort whose first follow-up occurred during the autumn of 2019 (before the pandemic). One possible explanation for this pattern of decreasing differences between exposed and unexposed is that loneliness and poor sleep may not be constant over time. Some participants who experienced loneliness and sleep problems at baseline may no longer have these problems at follow-up. Mental health symptoms over time dichotomized by pre-pandemic mental health problems were most likely subject to ‘regression towards the mean’. Our interpretation is that the groups exposed to pre-pandemic loneliness, poor sleep quality or mental health problems did not show unfavourable mental health trajectories during the first months of the COVID-19 pandemic compared to the unexposed groups.

Our results contrast with previous studies which reported that mental health problems worsened during the COVID-19 pandemic [4, 12]. However, our results agree with the results of Fancourt et al. [5] who showed that in a large UK cohort levels of depression and anxiety decreased during lockdown [5]. Our results may differ from previous research for several reasons including differences in study populations, differences in the spread of the virus between countries and differences in public-health strategies used to mitigate transmission of the SARS-CoV-2. Sweden’s public-health strategy was less intrusive than that of many other countries, which may have had less detrimental effects on mental health. Stockholm has had high mortality compared to other large cities, which one might expect to lead to more negative feelings and worry in relation to the pandemic; and thus, this does not explain why our results show less mental health impact than many other studies. More high-quality research is needed to compare mental health during the COVID-19 pandemic between different countries and populations, and also to evaluate long-term mental health effects of the pandemic and different societal strategies for managing it.

The strengths of our study include the conduct of a natural experiment to investigate differences in mental health symptoms before and after the pandemic reached Sweden. Secondly, the instruments used for measurements of all variables have good psychometric properties, limiting the risk of misclassification. Finally, we included a large and diverse sample of university students from six universities.

We recruited 27% of eligible students. Therefore, it is possible that selection bias influenced our results. However, the baseline pre-pandemic levels of mental health symptoms measured in our cohort were similar to those reported in previous studies of Swedish university students using the same instrument (DASS-21) [24]. This suggests that our sample was representative of the mental health status of Swedish university students before the pandemic. The follow-up response rate was 74% during the first follow-up period and 60% during the second follow-up period. It is possible that attrition of participants may have biased our estimates. However, our complete-case sensitivity analyses suggest that baseline differences between drop-outs and completers did not meaningfully affect the results. Our study sample was restricted to students from universities in Stockholm and was largely comprised of medical students, of whom most were in their first year of university education and the majority were women. The gender representation of the sample is very similar to that of Swedish medical students overall, but not of Swedish university students generally [31]. It is possible that our findings do not generalize well to students from other parts of Sweden, or students in other education programmes. That said, we believe that the challenges faced by the students in our sample were similar to those of Swedish university students overall during the COVID-19 pandemic (e.g. online education, reduced social contacts, etc.).

## Conclusions

Symptoms of depression, anxiety and stress among Swedish university students were stable during the first three months of the pandemic, while there were decreased symptom levels during the summer months, probably due to seasonality effects. Pre-pandemic loneliness, poor sleep quality or mental health problems were not associated with trajectories of depression, anxiety and stress symptoms. Contrary to our hypothesis, our results do not support a worsening of mental health in Swedish university students during the first months of the COVID-19 pandemic. Neither did we find worse trajectories for students exposed to pre-pandemic loneliness, poor sleep quality or mental health problems.

## Supplemental Material

sj-docx-1-sjp-10.1177_14034948211015814 – Supplemental material for Depression, anxiety and stress among Swedish university students before and during six months of the COVID-19 pandemic: A cohort study Click here for additional data file. Supplemental material, sj-docx-1-sjp-10.1177_14034948211015814 for Depression, anxiety and stress among Swedish university students before and during six months of the COVID-19 pandemic: A cohort study by Fred Johansson, Pierre Côté, Sheilah Hogg-Johnson, Ann Rudman, Lena w. Holm, Margreth Grotle, Irene Jensen, Tobias Sundberg, klara Edlund and Eva Skillgate in Scandinavian Journal of Public Health

sj-docx-2-sjp-10.1177_14034948211015814 – Supplemental material for Depression, anxiety and stress among Swedish university students before and during six months of the COVID-19 pandemic: A cohort study Click here for additional data file. Supplemental material, sj-docx-2-sjp-10.1177_14034948211015814 for Depression, anxiety and stress among Swedish university students before and during six months of the COVID-19 pandemic: A cohort study by Fred Johansson, Pierre Côté, Sheilah Hogg-Johnson, Ann Rudman, Lena w. Holm, Margreth Grotle, Irene Jensen, Tobias Sundberg, klara Edlund and Eva Skillgate in Scandinavian Journal of Public Health

sj-jpg-1-sjp-10.1177_14034948211015814 – Supplemental material for Depression, anxiety and stress among Swedish university students before and during six months of the COVID-19 pandemic: A cohort study Click here for additional data file. Supplemental material, sj-jpg-1-sjp-10.1177_14034948211015814 for Depression, anxiety and stress among Swedish university students before and during six months of the COVID-19 pandemic: A cohort study by Fred Johansson, Pierre Côté, Sheilah Hogg-Johnson, Ann Rudman, Lena w. Holm, Margreth Grotle, Irene Jensen, Tobias Sundberg, klara Edlund and Eva Skillgate in Scandinavian Journal of Public Health

sj-pptx-1-sjp-10.1177_14034948211015814 – Supplemental material for Depression, anxiety and stress among Swedish university students before and during six months of the COVID-19 pandemic: A cohort study Click here for additional data file. Supplemental material, sj-pptx-1-sjp-10.1177_14034948211015814 for Depression, anxiety and stress among Swedish university students before and during six months of the COVID-19 pandemic: A cohort study by Fred Johansson, Pierre Côté, Sheilah Hogg-Johnson, Ann Rudman, Lena w. Holm, Margreth Grotle, Irene Jensen, Tobias Sundberg, klara Edlund and Eva Skillgate in Scandinavian Journal of Public Health

## References

[1] Holmes EA O’Connor RC Perry VH , et al. Multidisciplinary research priorities for the COVID-19 pandemic: a call for action for mental health science. Lancet Psychiatry 2020;7(6):547–560. 32304649 10.1016/S2215-0366(20)30168-1 PMC7159850

[2] Ibrahim AK Kelly SJ Adams CE , et al. A systematic review of studies of depression prevalence in university students. J Psychiatr Res 2013;47(3):391–400. 23260171 10.1016/j.jpsychires.2012.11.015

[3] Rotenstein LS Ramos MA Torre M , et al. Prevalence of depression, depressive symptoms, and suicidal ideation among medical students: A systematic review and meta-analysis. JAMA 2016;316(21):2214–2236. 27923088 10.1001/jama.2016.17324 PMC5613659

[4] Thombs BD Bonardi O Rice DB , et al. Curating evidence on mental health during COVID-19: A living systematic review. J Psychosom Res 2020;133:1101–1113. 10.1016/j.jpsychores.2020.110113 PMC7185913 32354463

[5] Fancourt D, Steptoe A and Bu F. Trajectories of anxiety and depressive symptoms during enforced isolation due to COVID-19 in England: A longitudinal observational study. Lancet Psychiatry 2020; 8:141–149. 33308420 10.1016/S2215-0366(20)30482-X PMC7820109

[6] Elmer T Mepham K Stadtfeld C. Students under lockdown: Comparisons of students’ social networks and mental health before and during the COVID-19 crisis in Switzerland. PLoS One 2020;15(7):e0236337. 32702065 10.1371/journal.pone.0236337 PMC7377438

[7] Huckins JF daSilva AW Wang W , et al. Mental health and behavior of college students during the early phases of the COVID-19 pandemic: Longitudinal smartphone and ecological momentary assessment study. J Med Internet Res 2020;22(6):e20185. 32519963 10.2196/20185 PMC7301687

[8] Fried EI Papanikolaou F Epskamp S. Mental health and social contact during the COVID-19 pandemic: An ecological momentary assessment study. PsyArXiv 2020. DOI: 10.31234/osf.io/36xkp

[9] MQ: Transforming Mental Health and the Academy of Medical Sciences. Survey results: Understanding people’s concerns about the mental health impacts of the COVID-19 pandemic. The Academy of Medical Sciences, https://acmedsci.ac.uk/file-download/99436893 (2020, accessed 10 December 2020).

[10] Cacioppo JT Hawkley LC Thisted RA. Perceived social isolation makes me sad: 5-year cross-lagged analyses of loneliness and depressive symptomatology in the Chicago Health, Aging, and Social Relations Study. Psychol Aging 2010;25(2):453–463. 20545429 10.1037/a0017216 PMC2922929

[11] Wang J Mann F Lloyd-Evans B , et al. Associations between loneliness and perceived social support and outcomes of mental health problems: A systematic review. BMC Psychiatry 2018;18(1):156. 29843662 10.1186/s12888-018-1736-5 PMC5975705

[12] Vindegaard N Eriksen Benros M. COVID-19 pandemic and mental health consequences: Systematic review of the current evidence. Brain Behav Immun 2020;89:331–542. 10.1016/j.bbi.2020.05.048 PMC7260522 32485289

[13] Sivertsen B Vedaa Ø Harvey AG , et al. Sleep patterns and insomnia in young adults: A national survey of Norwegian university students. J Sleep Res 2019;28(2):e12790. 30515935 10.1111/jsr.12790

[14] Franceschini C Musetti A Zenesini C , et al. Poor sleep quality and its consequences on mental health during the COVID-19 lockdown in Italy. Front Psychol 2020;11:574475. 33304294 10.3389/fpsyg.2020.574475 PMC7693628

[15] Walker MP. The role of sleep in cognition and emotion. Ann NY Acad Sci 2009;1156:168–197. 19338508 10.1111/j.1749-6632.2009.04416.x

[16] Zohar D Tzischinsky O Epstein R , et al. The effects of sleep loss on medical residents’ emotional reactions to work events: A cognitive-energy model. Sleep 2005;28(1):47–54. 15700720 10.1093/sleep/28.1.47

[17] Yao H Chen JH Xu YF. Patients with mental health disorders in the COVID-19 epidemic. Lancet Psychiatry 2020;7(4):e21. 32199510 10.1016/S2215-0366(20)30090-0 PMC7269717

[18] Cuijpers P Smit F. Subthreshold depression as a risk indicator for major depressive disorder: a systematic review of prospective studies. Acta Psychiatr Scand 2004;109(5):325–331. 15049768 10.1111/j.1600-0447.2004.00301.x

[19] Lee YY Stockings EA Harris MG , et al. The risk of developing major depression among individuals with subthreshold depression: a systematic review and meta-analysis of longitudinal cohort studies. Psychol Med 2019;49(1):92–102. 29530112 10.1017/S0033291718000557

[20] The Public Health Agency of Sweden. Ny fas kräver nya insatser mot covid-19, https://www.folkhalsomyndigheten.se/nyheter-och-press/nyhetsarkiv/2020/mars/ny-fas-kraver-nya-insatser-mot-covid-19/ (2020, accessed 23 December 2020).

[21] The Public Health Agency of Sweden. Lärosäten och gymnasieskolor uppmanas nu att bedriva distansundervisning, https://www.folkhalsomyndigheten.se/nyheter-och-press/nyhetsarkiv/2020/mars/larosaten-och-gymnasieskolor-uppmanas-nu-att-bedriva-distansundervisning/ (2020, accessed 23 December 2020).

[22] The Public Health Agency of Sweden. Beteende, oro och informationsbehov: Genomförda och pågående undersökningar under covid-19. Report, The Public Health Agency of Sweden,No. 20101, 24 June 2020.

[23] Henry JD Crawford JR. The short-form version of the Depression Anxiety Stress Scales (DASS-21): Construct validity and normative data in a large non-clinical sample. Br J Clin Psychol. 2005;44(Pt 2):227–239 16004657 10.1348/014466505X29657

[24] Alfonsson S Wallin E Maathz P. Factor structure and validity of the Depression, Anxiety and Stress Scale-21 in Swedish translation. J Psychiatr Ment Health Nurs 2017;24(2–3):154–162. 28124410 10.1111/jpm.12363

[25] Lovibond S Lovibond P. Manual for the Depression Anxiety Stress Scales (DASS). Psychology Foundation Monograph. Sydney: University of New South Wales, 1993.

[26] Ronk FR Korman JR Hooke GR , et al. Assessing clinical significance of treatment outcomes using the DASS-21. Psychol Assess 2013;25(4):1103–1110. 23730826 10.1037/a0033100

[27] Hughes ME Waite LJ Hawkley LC , et al. A short scale for measuring loneliness in large surveys: Results from two population-based studies. Res Aging 2004;26(6):655–672. 18504506 10.1177/0164027504268574 PMC2394670

[28] Steptoe A Shankar A Demakakos P , et al. Social isolation, loneliness, and all-cause mortality in older men and women. Proc Natl Acad Sci USA 2013;110(15):5797–5801. 23530191 10.1073/pnas.1219686110 PMC3625264

[29] Carpenter JS Andrykowski MA. Psychometric evaluation of the Pittsburgh Sleep Quality Index. J Psychosom Res 1998;45(1):5–13. 9720850 10.1016/s0022-3999(97)00298-5

[30] Buysse DJ Reynolds CF 3rd Monk TH , et al. The Pittsburgh Sleep Quality Index: A new instrument for psychiatric practice and research. Psychiatry Res 1989;28(2):193–213. 2748771 10.1016/0165-1781(89)90047-4

[31] Universitetskanslerämbetet. Könsuppdelningen bland de examinerade i högskolan består. Report No.: 2019-03-26 /5. https://www.uka.se/download/18.9bcba6c169953c27d41a67/1553595611137/statistisk-analys-2019-03-26-konsuppdelning-bland-de-examinerade-i-hogskolan-bestar.pdf (2019, accessed 10 March 2021).

