# Supplementary material for: Depression, anxiety and stress among Swedish university students before and during six months of the COVID-19 pandemic: A cohort study
Source: Scand J Public Health. 2021 May 26;49(7):741–9. doi: 10.1177/14034948211015814 (PMC8521369; doi:10.1177/14034948211015814)
Supplement: sj-docx-1-sjp-10.1177_14034948211015814 – Supplemental material for Depression, anxiety and stress among Swedish university students before and during six months of the COVID-19 pandemic: A cohort study [file sj-docx-1-sjp-10.1177_14034948211015814.docx]

0

5

10

15

20

Aug-Sep

Nov-Jan

Time-Period

Depression score

L

0

1

0

5

10

15

20

Aug-Sep

Nov-Jan

Time-Period

Anxiety score

L

0

1

0

5

10

15

20

Aug-Sep

Nov-Jan

Time-Period

Stress score

Loneliness

Not lonely

Lonely

0

5

10

15

20

Aug-Sep

Nov-Jan

Time-Period

Depression score

S

0

1

0

5

10

15

20

Aug-Sep

Nov-Jan

Time-Period

Anxiety score

S

0

1

0

5

10

15

20

Aug-Sep

Nov-Jan

Time-Period

Stress score

Sleep quality

Good

Poor

0

5

10

15

20

Aug-Sep

Nov-Jan

Time-Period

Depression score

P

0

1

0

5

10

15

20

Aug-Sep

Nov-Jan

Time-Period

Anxiety score

P

0

1

0

5

10

15

20

Aug-Sep

Nov-Jan

Time -Period

Stress score

Pre-existing

MH problems

No

Yes

**eFigure 2. Differences in DASS-21 scores from Aug-Sep 2019 to Nov 2019-Jan 2020.** Estimated means from GEE models specified as in main analyses. Participants are students in the SUN-study recruited from August-September 2019 (n=494) with 1^st^ follow-up pre-pandemic, between November 2019 and January 2020 (n=400). Participants are from Sophiahemmet University and The Scandinavian College of Naprapathic Manual Medicine. At baseline 70.2 % were women and the mean age was 27.7 years.
