# Supplementary material for: Depression, anxiety and stress among Swedish university students before and during six months of the COVID-19 pandemic: A cohort study
Source: Scand J Public Health. 2021 May 26;49(7):741–9. doi: 10.1177/14034948211015814 (PMC8521369; doi:10.1177/14034948211015814)
Supplement: sj-docx-2-sjp-10.1177_14034948211015814 – Supplemental material for Depression, anxiety and stress among Swedish university students before and during six months of the COVID-19 pandemic: A cohort study [file sj-docx-2-sjp-10.1177_14034948211015814.docx]

eTable 1. Complete cases (n=1049) GEE-model coefficients on mean level depression, anxiety and stress symptoms over the three time-periods for complete cases only.

|  | Depression  Coefficient (95% CI) | Anxiety  Coefficient (95% CI,) | Stress  Coefficient (95% CI) |
| --- | --- | --- | --- |
| Model with only time-period  FU1  FU2 | 0.32 (0.05 to 0.55)  -0.62 (-0.89 to -0.37) | -0.08 (-0.25 to -0.09)  -0.71 (-0.89 to -0.53) | -0.26 (-0.50 to -0.01)  -1.22 (-1.48 to -0.97) |
| Model with loneliness and time-period  Loneliness  FU1  FU2  Loneliness * FU1  Loneliness * FU2 | 4.00 (3.45 to 4.56)  0.65 (0.37 to 0.92)  -0.18 (-0.46 to 0.09)  -0.89 (-1.40 to -0.38)  -1.17 (-1.69 to -0.65) | 2.26 (1.84 to 2.69)  0.22 (0.04 to 0.41)  -0.36 (-0.55 to -0.17)  -0.81 (-1.19 to -0.44)  -0.95 (-1.35 to -0.55) | 3.21 (2.67 to 3.74)  0.11 (-0.18 to 0.40)  -1.01 (-1.31 to -0.71)  -0.99 (-1.52 to -0.46)  -0.57 (-1.12 to -0.01) |
| Model with sleep quality and time-period  Poor sleep quality  FU1  FU2  Poor sleep quality* FU1  Poor sleep quality * FU2 | 3.60 (3.12 to 4.08)  0.53 (0.25 to 0.81)  -0.19 (-0.47 to 0.08)  -0.42 (-0.88 to 0.04)  -0.81 (-1.28 to -0.35) | 2.26 (1.89 to 2.62)  0.12 (-0.09 to 0.33)  -0.39 (-0.60 to -0.17)  -0.37 (-0.70 to -0.04)  -0.61 (-0.96 to -0.26) | 3.51 (3.02 to 4.01)  0.03 (-0.31 to 0.38)  -0.82 (-1.15 to -0.48)  -0.55 (-1.04 to -0.05)  -0.80 (-1.30 to -0.29) |
| Model with PPMHP and time-period  PPMHP  FU1  FU2  PPMHP * FU1  PPMHP * FU2 | 6.51 (6.02 to 7.01)  1.13 (0.91 to 1.35)  0.34 (0.12 to 0.56)  -2.17 (-2.70 to -1.64)  -2.57 (-3.11 to -2.03) | 4.35 (3.96 to 4.74)  0.48 (0.32 to 0.64)  0.03 (-0.12 to 0.18)  -1.49 (-1.87 to -1.10)  -1.98 (-2.39 to -1.57) | 6.45 (6.00 to 6.90)  0.60 (0.34 to 0.86)  -0.28 (-0.53 to -0.03)  -2.30 (-2.83 to -1.76)  -2.53 (-3.10 to -1.96) |

All models, except the ones with only time as predictor, were adjusted for gender (female vs male and other) and age (continuous scale). FU1: First follow-up period, FU2 Second follow-up period, PPMHP: Pre-Pandemic Mental Health Problems.
