# Supplementary figures and images for: Depression, anxiety and stress among Swedish university students before and during six months of the COVID-19 pandemic: A cohort study
Source: Scand J Public Health. 2021 May 26;49(7):741–9. doi: 10.1177/14034948211015814 (PMC8521369; doi:10.1177/14034948211015814)

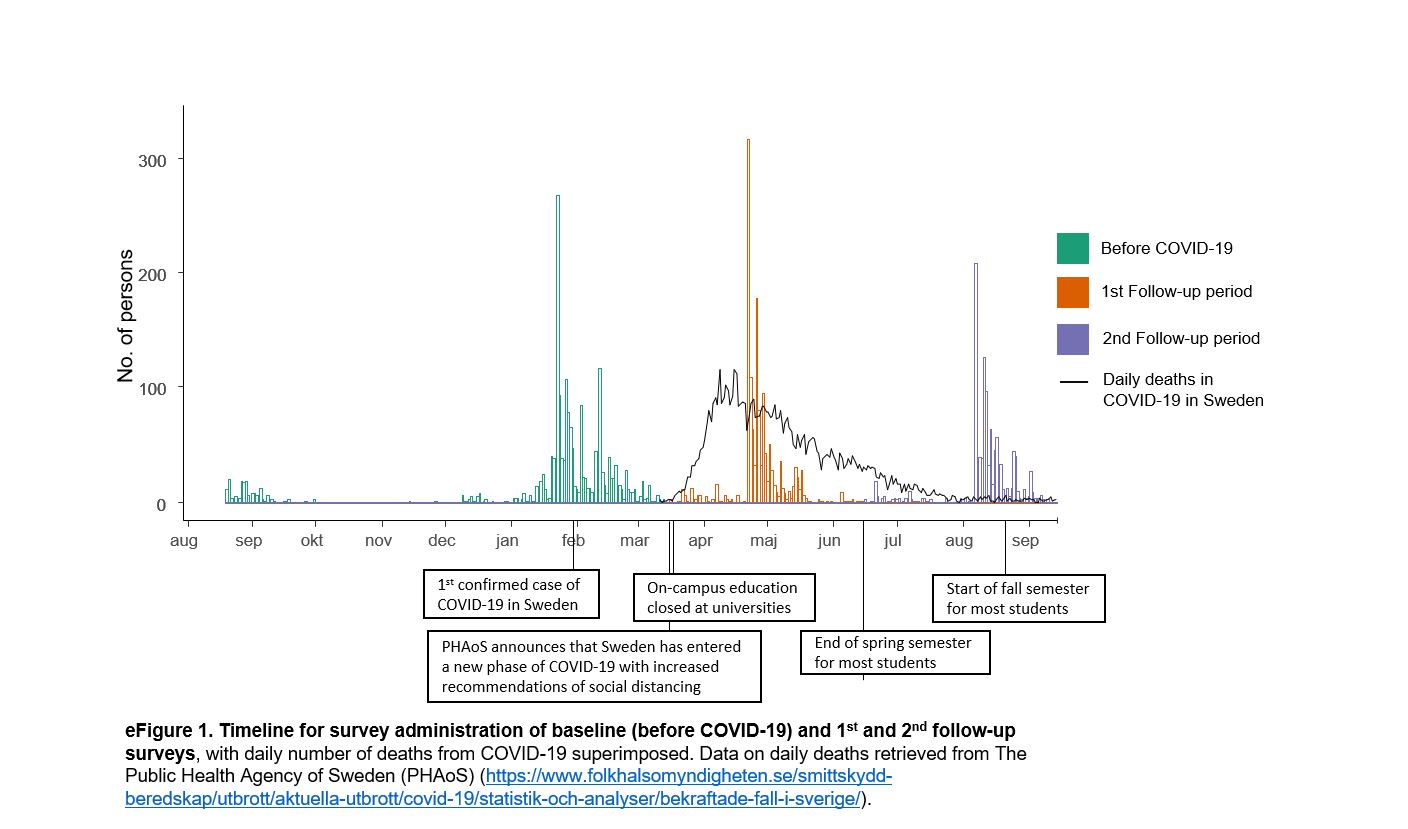

Supplement: sj-jpg-1-sjp-10.1177_14034948211015814 – Supplemental material for Depression, anxiety and stress among Swedish university students before and during six months of the COVID-19 pandemic: A cohort study [file sj-jpg-1-sjp-10.1177_14034948211015814.JPG]
