# Supplementary material for: Depression, anxiety and stress among Swedish university students before and during six months of the COVID-19 pandemic: A cohort study
Source: Scand J Public Health. 2021 May 26;49(7):741–9. doi: 10.1177/14034948211015814 (PMC8521369; doi:10.1177/14034948211015814)
Supplement: sj-pptx-1-sjp-10.1177_14034948211015814 – Supplemental material for Depression, anxiety and stress among Swedish university students before and during six months of the COVID-19 pandemic: A cohort study [file sj-pptx-1-sjp-10.1177_14034948211015814.pptx]

## Slide 1
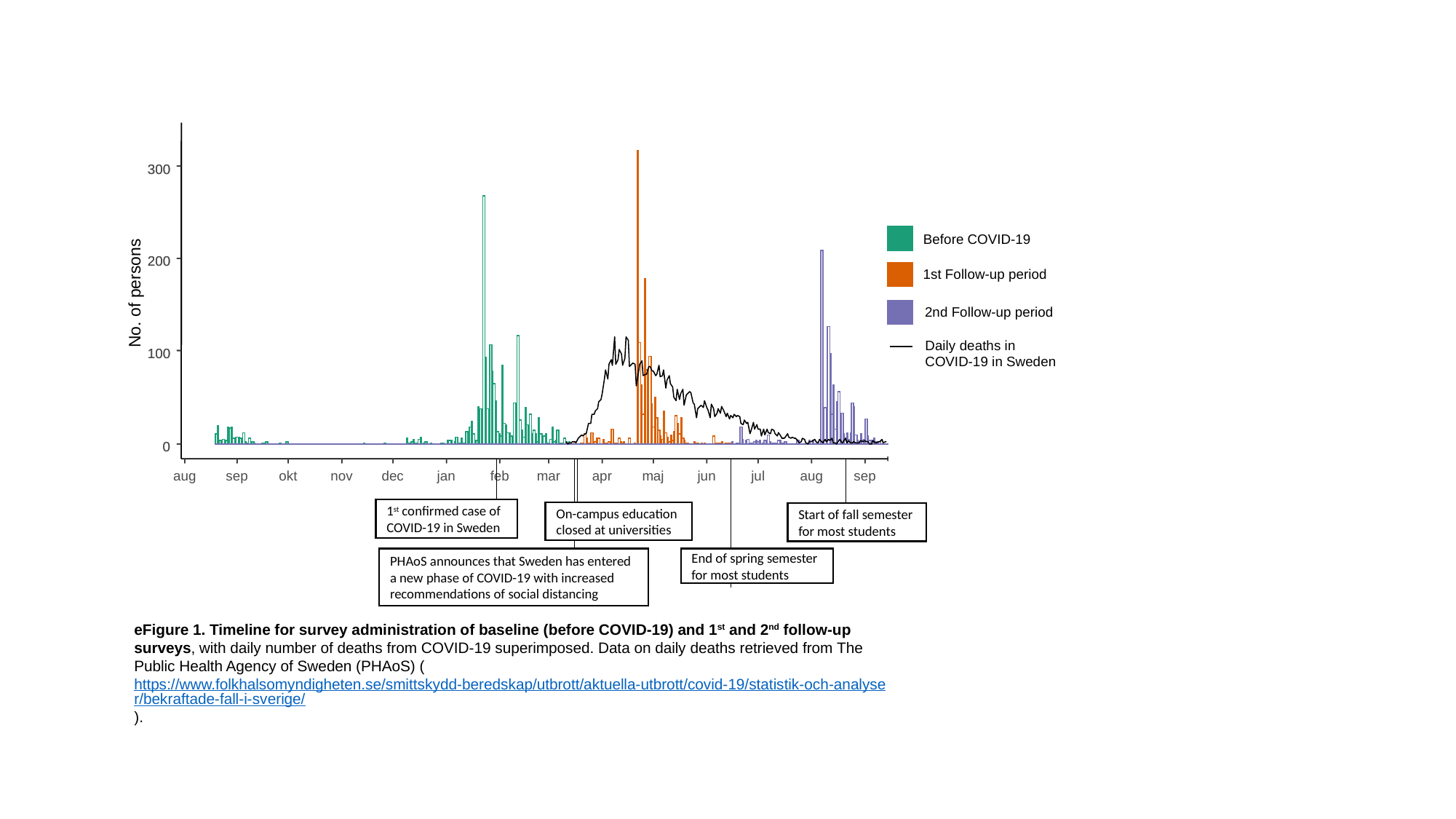

300
200
100
0
aug
sep
okt
nov
dec
jan
feb
mar
apr
maj
jun
jul
aug
sep
300
200
No. of persons
100
0
Before COVID-19
1st Follow-up period
2nd Follow-up period
Daily deaths in
COVID-19 in Sweden
1st confirmed case of COVID-19 in Sweden
On-campus education closed at universities
Start of fall semester for most students
PHAoS announces that Sweden has entered a new phase of COVID-19 with increased recommendations of social distancing
End of spring semester for most students
eFigure 1. Timeline for survey administration of baseline (before COVID-19) and 1st and 2nd follow-up surveys, with daily number of deaths from COVID-19 superimposed. Data on daily deaths retrieved from The Public Health Agency of Sweden (PHAoS) (https://www.folkhalsomyndigheten.se/smittskydd-beredskap/utbrott/aktuella-utbrott/covid-19/statistik-och-analyser/bekraftade-fall-i-sverige/).
